# Goal-Oriented Physiotherapy Protocol of a 55-Year Male With Squamous Cell Carcinoma of the Tongue: A Case Report

**DOI:** 10.7759/cureus.30680

**Published:** 2022-10-25

**Authors:** Vaishnavi B Warutkar, Shubhangi Patil, Simran Y Jaiswal

**Affiliations:** 1 Community Health Physiotherapy, Ravi Nair Physiotherapy College, Datta Meghe Institute of Medical Sciences (Deemed to be University), Wardha, IND

**Keywords:** oral cancer, physiotherapy, mouth gauge, swallowing exercise, carcinoma of the tongue

## Abstract

Although oral cancer is well-known, the occurrence rate of the disease varies greatly globally. Squamous cell carcinoma of the tongue, which frequently starts in the lateral tongue, is the most common kind of oral cancer. In this instance, a male patient was found to have squamous cell carcinoma of the tongue and had undergone surgery. Following any surgical treatment of the tongue, discomfort, septicemia, difficulty eating, and speech issues are the most common oral consequences. His chief complaints were pain and difficulty in mouth opening. He also had the inability to move his tongue, which made talking and swallowing difficult. He had a history of chewing tobacco and smoking cigarettes for the past 15 years. A physiotherapy program was advised to the patient to reduce his symptoms, which included mouth-opening exercises, swallowing exercises, etc. The case's findings indicate that, when compared to the outcome measures, all objectives were met, and the patient improved in his activities of daily living.

## Introduction

The oral cavity is usually regarded as the sixth to the ninth most widespread area of the body for carcinoma, based mainly on the country (and also geographic location in some nations) and gender of the patient population. Oral cancer is widely known, but the incidence rate of cancer differs widely around the world [1]. It is unusual in that, although having a low overall incidence which is nearly 1% compared to other malignancies, its occurrence has been increasing over the past 50 years [2]. The most typical oral cancer is squamous cell carcinoma of the tongue, which often begins in the lateral tongue. It often affects males in their middle years. Two risk factors are consuming alcohol and smoking. Individuals may complain of pain or burning in the tongue [3,4]. Elderly people frequently have the highest rates of head and neck tumours. This is a result of the normal pattern of neoplastic origin and development that occurs in these anatomical locations, which is marked by an incremental accumulation of mutations that eventually results in a full-blown neoplastic transformation. In this way, the adult-elderly age is crucial for enabling the accumulation of genetic changes at a rate that is conducive to malignancy [5].

The most frequent oral complications following any surgical procedure are pain, septicemia, eating difficulties, speaking problems, etc. Surgical oncology's cornerstone principle is to clear the main tumour while preserving a wide margin of normal tissue in its place [6].

This case is of a male patient who was diagnosed with differential squamous cell carcinoma of the tongue and was further operated on for subtotal glossectomy with radial forearm flap reconstruction. The form of the subsequent dysfunction is dependent on a few other variables. The extent of oral tongue and tongue base removal leads to swallowing difficulty [7]. Even if surgery aids to eliminate the malignant carcinoma, the individual is left with structural abnormalities, stitches and scarring, edema, a decrease in psychological and social confidence, as well as other adverse consequences that we refer to as surgical affectability [8]. A thorough rehabilitation program was necessary to minimize the repercussions of the operations and improve his quality of life.

## Case presentation

A 55-year-old male patient was brought to our inpatient department. The patient was apparently alright six months back when he noticed an ulcer on the right lateral border of the tongue. The patient previously went to a local hospital, where an X-ray was done and then he was referred to our hospital for further treatment. contrast-enhanced computed tomography (CECT) and MRI were done and the MRI tongue revealed that an ill-defined heterogeneously enhancing mass was noted in the lateral border of the oral tongue; the patient was also advised to do a histopathological correlation, which revealed that the given tissue piece showed one lymph node and was suggestive of reactive lymphadenitis and was positive for infiltration by malignant epithelial cells.

The ulcer was insidious in onset, gradually progressive in nature, initially smaller in size, now progressed to a larger size, associated with difficulty in swallowing, both solids and liquids, associated with pain, throbbing type, intermittent, radiating to the back of neck and head on the right side, no aggravating factors, and partially relieved with medication. He also underwent two cycles of chemotherapy.

The patient had a history of loss of appetite, he also lost weight by 5-7 kg. He had a history of consuming tobacco and cigarette smoking for 15 years. There was no history of addictions at the time of the presentation. The patient was admitted to the hospital on August 18, 2022. His surgery was done on August 23 and then physiotherapy was started on August 25. 

Clinical findings

Prior to the examination, proper consent of the patient was taken. The patient's post-operative day two examination is given in Table 1.

**Table 1:** Patient examination post-operative day 2

Variable	Movement	Post-operative day 1	
Range of motion- Cervical joint	Flexion	0-45^0^	
	Extension	0-40^0^	
		Right side	Left side
	Lateral flexion	0-35^0^	0-35^0^
Temporomandibular joint (centimeters)	Mouth opening was 1.5cm present.		
Manual muscle testing	Cervical flexors	2/5	2/5
	Cervical extensors	2/5	2/5
	Side flexors	3/5	2/5
	Shoulder flexors	3/5	2/5
	Shoulder extensors	4/5	2/5
	Abductors	3/5	2/5
	Adductors	3/5	2/5
	Medial rotators	2/5	2/5
	Lateral rotators	2/5	2/5

Pain Assessment:

The grade of tenderness was 3 (patient winces and withdraws). On the numerical pain rating scale, the score was 7/10 on rest and 9/10 on movement.

Medical management

He underwent surgery for a right subtotal glossectomy with composite resection with bilateral neck dissection with radial forearm flap reconstruction with tracheostomy and postoperative medications taken in the hospital were tablet Pan 40mg twice daily for seven days, tablet Ondem 4mg twice a day for seven days, chlorhexidine mouthwash, syrup Duphalac 30ml, tablet Stemetil 5mg.

Physiotherapy management

**Figure 1:**
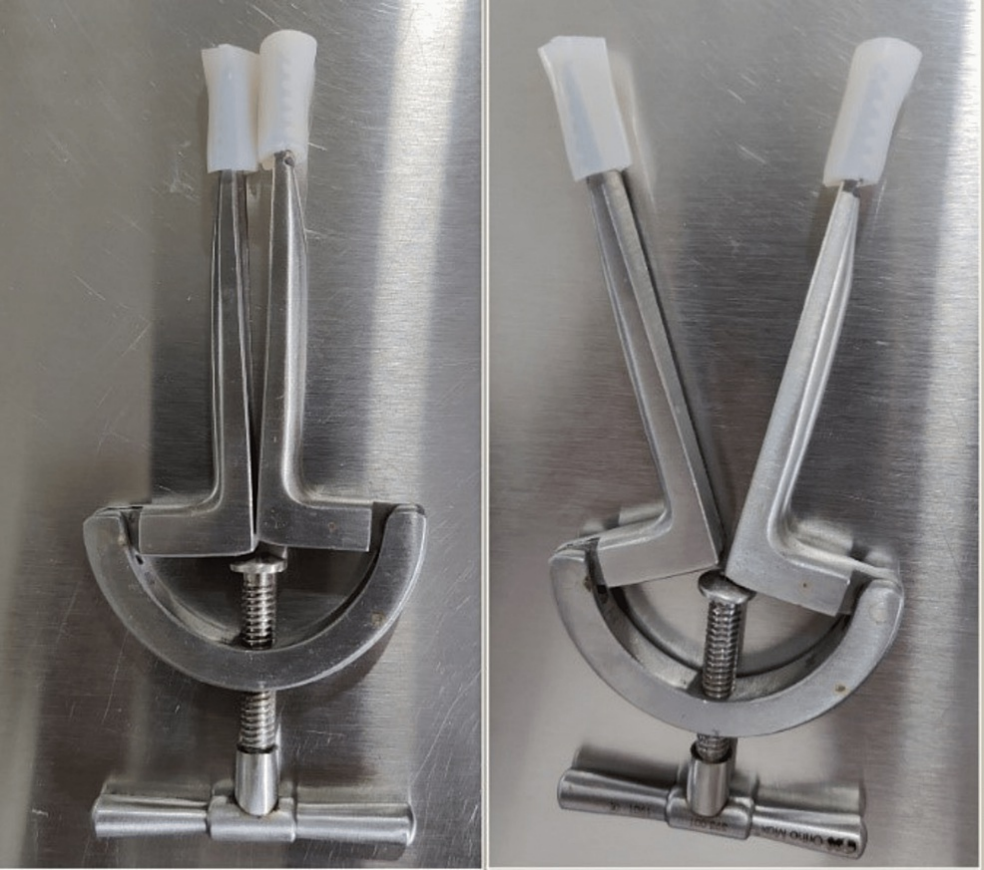
Mouth gauge

**Figure 2:**
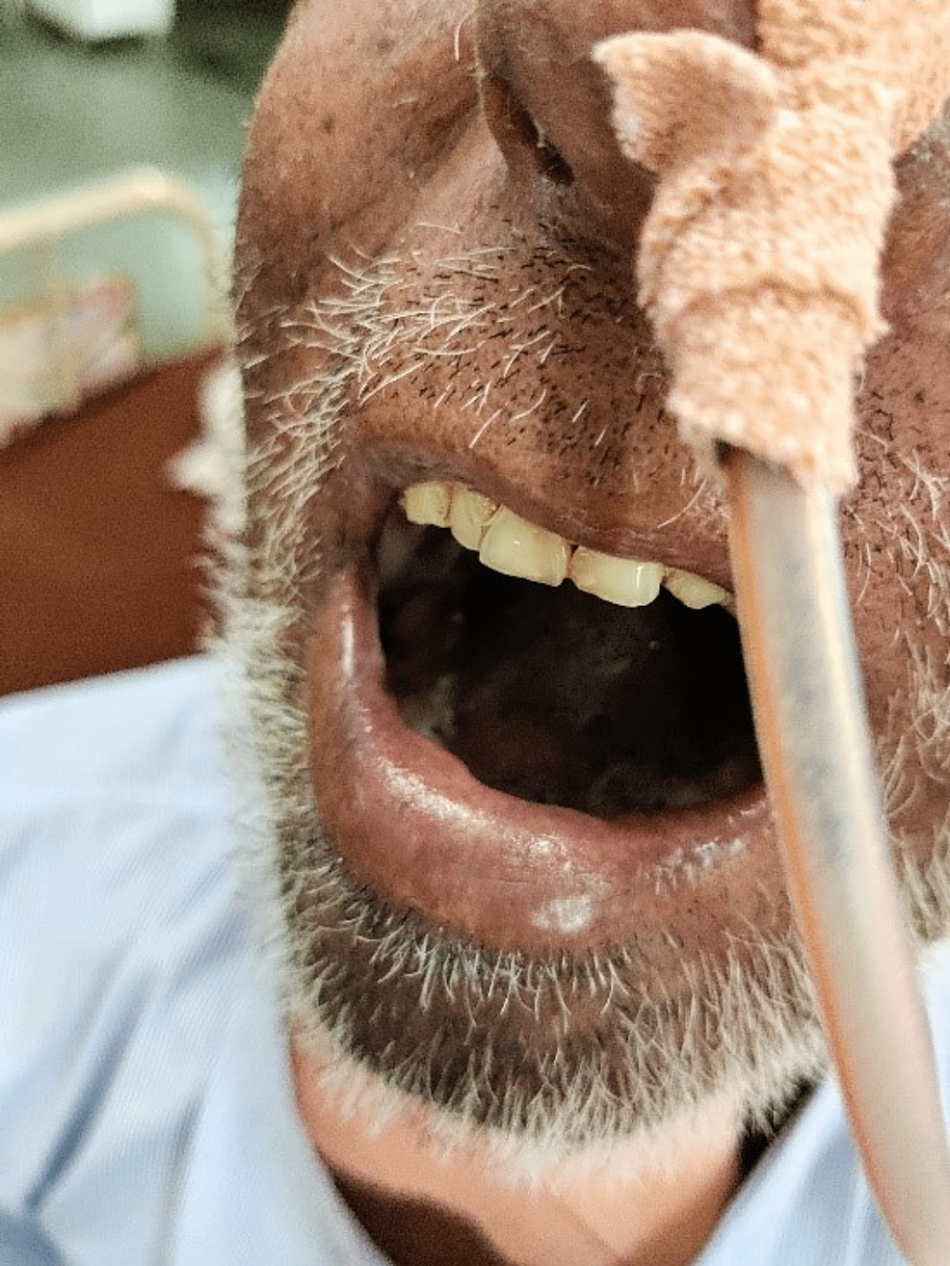
Mouth-opening post physiotherapy

**Figure 3:**
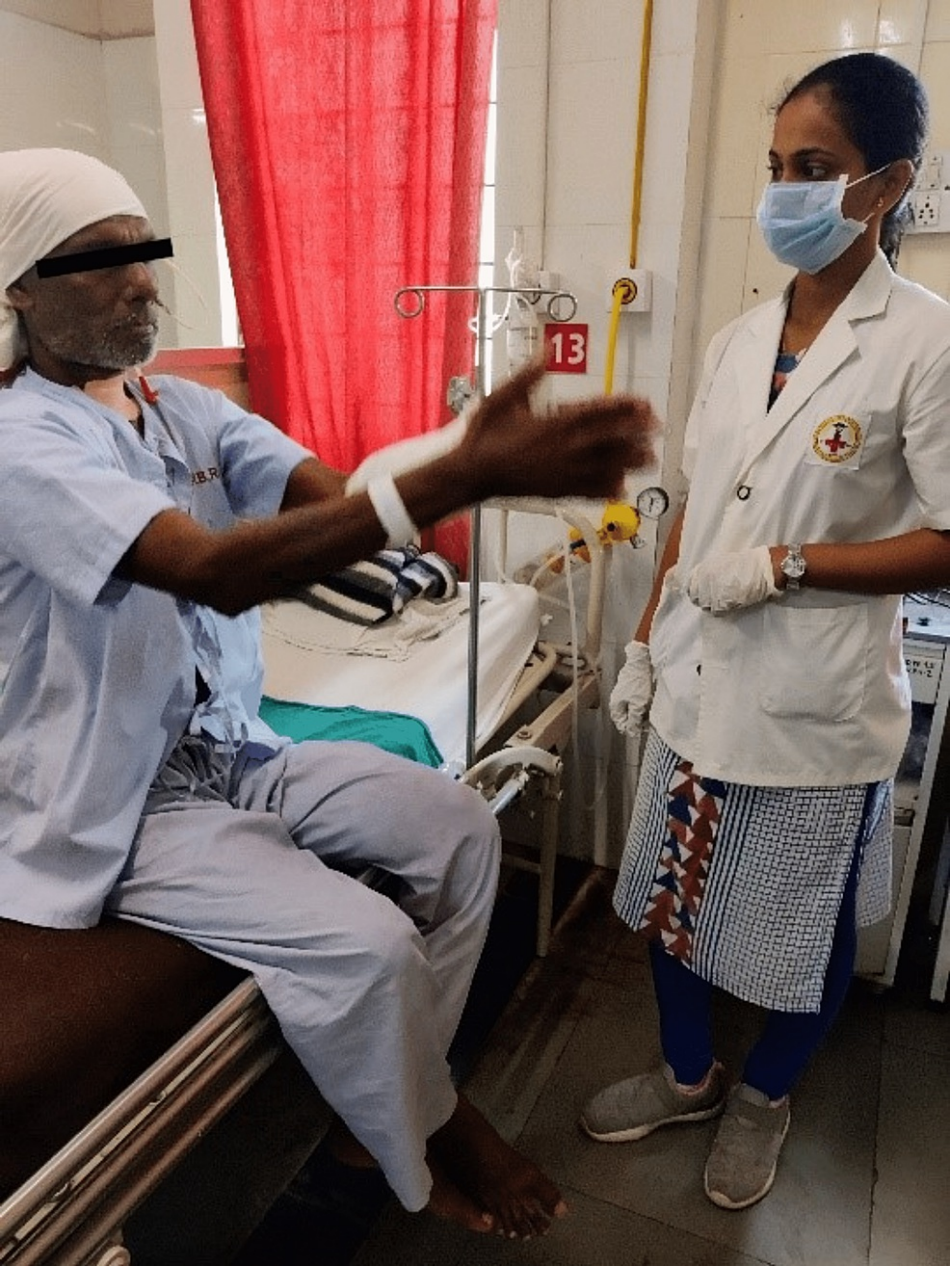
Patient while doing thoracic expansion exercise

The goal-oriented protocol detailed in Table 2 was from postoperative day 2 to postoperative day 9. A mouth gauge was used to help with mouth-opening exercises (Figure 1, 2). Besides other exercises, thoracic exercises were done to maintain the functional capacity of the lungs (Figure 3).

**Table 2:** Physiotherapy treatment protocol, Week 1 (days 2-9)

Goal	Intervention	Duration
To begin mouth opening	Active range of motion for temporomandibular joint in all planes; chin tuck exercises; mouth gauge.	10 repetitions, 3 times per day. The mouth gauge should be used 5-6 times a day.
To enhance tongue movements	Tongue motions in all directions (tongue protrusion, tongue retraction, tongue elevation, tongue side to side).	Can be carried out as frequently as feasible during the day.
To improve the range of motion (ROM) of the cervical joint.	Cervical range of motion exercises.	10 reps, 3 times per day
To maintain strength	Cervical Isometric strengthening exercises; upper-limb strengthening using a 1/2 litre water bottle.	10 reps, 3 times per day
To maintain the functional capacity of the lungs.	Deep breathing exercises; thoracic expansion exercises (with a 3-second hold).	10 reps, 3 times per day
To prevent edema, pressure ulcer, and improve circulation	Ankle pumps.	15 reps, 3 times per day

Home Exercise Program (HEP)

The patient was discharged on August 31. He was asked to continue all the above exercises at home as part of the HEP. He was also advised to do the following exercises: (i) Bubble-blowing exercise; (ii) Water holding exercise: the patient was asked to hold water in his mouth for 10 seconds and release it; (iv) Self-resisting exercise for the mouth: the patient was asked to open their mouth and insert his thumb to one side and then close and divert his mouth to the opposite side and hold for 10 seconds and then release.

Results

Pain Assessment:

The grade of tenderness was 1 (mild tenderness to palpation). On the numerical pain rating scale, the score was 3/10 on rest and 5/10 on movement. Patient examination details post-operative day 9 are given in Table 3.

**Table 3:** Patient examination post-operative day 9

Variable	Movement	Post-Op Day 7	
Range of motion- Cervical joint	Flexion	0-60^0^	
	Extension	0-75^0^	
		Right side	Left side
	Lateral flexion	0-45^0^	0-45^0^
Temporomandibular joint (centimeters)	The mouth opening was 4cm. (Figure 1)		
Manual muscle testing	Cervical flexors	3/5	3/5
	Cervical extensors	3/5	3/5
	Side flexors	3+/5	3+/5
	Shoulder flexors	3+/5	3+/5
	Shoulder extensors	3+/5	3+/5
	Abductors	3+/5	3+/5
	Adductors	3/5	3/5
	Medial rotators	3+/5	3+/5
	Lateral rotators	3/5	3/5

## Discussion

This case report describes a 55-year-old patient's complete rehabilitation protocol for one week after undergoing surgery for right subtotal glossectomy with composite resection with bilateral neck dissection with radial forearm flap reconstruction. The patient showed improvement in the one week of rehabilitation. The patient was discharged after a week and to maintain and improve his condition further, he was given a HEP.

Because of several interventions, including operations, chemotherapy, and radiation therapy, cancer victims experience a higher risk of mortality. The much more typical kind of mouth cancer is tongue-based squamous cell carcinoma. Zuydam et al. ascribe this to the tongue's crucial contribution to the bolus's movement between the mouth and throat, in addition to the tongue base's significant contribution to the generation of force in the pharynx [7]. Difficulties in swallowing can result from the removal of even very minor tumours that surround the base of the tongue [7,9]. In our case, the patient's restricted tongue mobility, which made talking and swallowing challenging, was one of his main problems. As per the study of Saifee et al. exercises for the tongue were advised, including tongue protrusion, retraction, the tongue-hold manoeuvre, range of motion, and strength training [8]. These exercises improved the tongue's musculature's movement and agility, which in turn helped to shape the tongue to integrate speech. Training to retain range of motion in the labial, lingual, jaw, neck, mandibular, and shoulder as well as jaw resistance methods were used as therapeutic procedures [8,10].

Individuals with malignancy who get different therapies, such as head and neck exercises, mouth opening and closing exercises, and shoulder movement activities, benefit greatly from physical therapy. Multiple problems brought on by cancer therapies are eliminated or treated. A postoperative physical therapy rehabilitation program aids these cancer victims in rebuilding their physical, mental, and social identities in order to regain adequate effective range of motion and so enhance their standard of living.

## Conclusions

This case report gives a detailed plan of a treatment protocol for individuals who are operated on for tongue carcinoma. The results of this case say that all goals were achieved when compared with the outcomes measures of pre-physiotherapy and post-physiotherapy treatment and the patient had shown improvement throughout in terms of speech, mouth opening, and swallowing. Physiotherapy is very important for post-operative patients with tongue carcinoma for faster recovery. Even a one-week physiotherapy exercise can be beneficial for advancing a patient's condition. It also decreases the risk of further complications, helps the patient become independent to do his routine everyday tasks, and improves his standard of living. 
